# Effective Moment Feature Vectors for Protein Domain Structures

**DOI:** 10.1371/journal.pone.0083788

**Published:** 2013-12-31

**Authors:** Jian-Yu Shi, Siu-Ming Yiu, Yan-Ning Zhang, Francis Yuk-Lun Chin

**Affiliations:** 1 School of Life Science, Northwestern Polytechnical University, Xi’an, Shaanxi Province, China; 2 Department of Computer Science, The University of Hong Kong, Hong Kong, China; 3 School of Computer Science, Northwestern Polytechnical University, Xi’an, Shaanxi Province, China; King’s College, London, United Kingdom

## Abstract

Imaging processing techniques have been shown to be useful in studying protein domain structures. The idea is to represent the pairwise distances of any two residues of the structure in a 2D distance matrix (DM). Features and/or submatrices are extracted from this DM to represent a domain. Existing approaches, however, may involve a large number of features (100–400) or complicated mathematical operations. Finding fewer but more effective features is always desirable. In this paper, based on some key observations on DMs, we are able to decompose a DM image into four basic binary images, each representing the structural characteristics of a fundamental secondary structure element (SSE) or a motif in the domain. Using the concept of moments in image processing, we further derive 45 structural features based on the four binary images. Together with 4 features extracted from the basic images, we represent the structure of a domain using 49 features. We show that our feature vectors can represent domain structures effectively in terms of the following. (1) We show a higher accuracy for domain classification. (2) We show a clear and consistent distribution of domains using our proposed structural vector space. (3) We are able to cluster the domains according to our moment features and demonstrate a relationship between structural variation and functional diversity.

## Introduction

The study of protein structures is no doubt a very important issue as structures are related to functions and can provide insights on the evolution of proteins. As protein structure is more conserved than its sequence throughout evolution, remote homologies should be detected in the universe of protein structures [1]. Proteins with similar structures are known to have similar functions. Thus, the classification of protein structures is a crucial step to capture functionality as well as evolutionary relationships of the proteins [2], [3]. In the following, we focus our discussion on protein domains which are the functional units of proteins [4].

A straightforward approach to do classification is to align and compare the 3D structures of two protein domains (e.g. CE [5] and Dali [6]). Based on a similarity score (e.g. root-mean-square deviation (RMSD)), we can decide if they are in the same class. However, there are several drawbacks to this approach. First, the score usually captures very little biological context of functions and evolution [4]. Second, 3D structural alignment is computation-intensive, and the score may violate the triangular inequality [7]. To resolve this problem, another direction is to perform feature extraction, a common method in pattern recognition and computer vision [8]. In this approach, important features related to the structure are extracted and represented as a feature vector. Instead of aligning the 3D structures directly, we compare the vectors. Since we do not need to align 3D structures, the comparison can be done much faster and can be used for all-against-all comparison, clustering and classification, and even structure retrieval with a well-designed indexing system in large scale datasets [9]. Moreover, the feature vectors, with the help of tools such as principal component analysis, enable researchers to analyze and visualize the distribution of domains in a 3-dimensional structural universe [3], [7], [10], [11] so as to deduce important factors related to evolution and functions.

There has been quite a lot of work following this feature extraction paradigm. Some characterize the domain structure directly as a topological object. Scaled Gauss Metric (SGM) [7] treated the protein backbone as a space curve, applied knot theory to describe such a curve by 29 topological invariants [12] and combined the length of domain into a 30-D feature vector for a domain. Recently, Penner et al. [11] exploited the idea of homeomorphism, a kind of topological isomorphism, to transform protein structure into a Fatgraph [13], and used two topological invariants of Fatgraph, the number of twisted alpha carbon linkages and the length of domain together as a feature vector of a domain structure. In general, topological characterization of domain demands complicated mathematical operations.

To speed up the feature extraction, as inspired from computer vision and pattern recognition, Choi et al. [10] considered the distance matrix (DM), a 2D matrix storing the pairwise distances of any two residues, as a representation of a domain structure. Different structures show different DMs. Simple thresholds were employed to discretize the image and 100 overlapping submatrices were selected from DM by partitioning around the medoids [14]. The frequencies of these submatrices were counted and grouped into a 100-D vector called local feature frequency profile (LFF). On the other hand, Chi et al. [15] treated a DM as a textural image, and extracted 24 features by local histograms as well as 9 features by Haralicks texture descriptors to represent a domain. But the extraction of the histogram and textural features of an image involves time-consuming computation. FragBag [16] was inspired by bag-of-word (BOW) in text recognition. It regarded protein structure as a BOW in which the words were 400 short fragments of the structure, counted the occurring numbers of all words in a protein by local structure alignment and combined them together into a 400-D feature vector as a representation of a domain structure. To summarize, these existing methods either use a large feature vector or involve complicated mathematical operations. Thus, representing domain structures with meaningful and short feature vectors remains a challenge.

Motivated by the previous findings that different types of structures show different images of DM and common types of structures show similar images [10], [15], in this paper, we also treat a DM as a textural image for effective and efficient analysis. We observe that, based on the inter-distance between residues (as given in DM) and the key patterns for different fundamental secondary structure elements (SSE), such as alpha helices and beta sheets, as well as basic structural motifs (e.g. 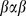 motif), different patterns exist in the DM image. Thus, using Gabor filter efficiently, we can decompose a DM into four binary contact images (BCM) representing alpha helix, parallel and anti-parallel beta sheet and other structure bondings. Each of these basic images represents the structural characteristics of one type of fundamental SSE or motifs. By using the concepts of moments in image processing, we calculate a series of image moments for each BCM from low order to high order (capturing structural characteristics from low to high granularity, such as the sizes of these SSEs and their inter-relationship). These moment series together with some simple counting statistics on BCM will form a 49-D feature vector (please refer to the Method section for a detailed description of our workflow). Since our feature vector is built from elementary SSE and motifs, it captures the structural information more effectively even with only 49 features.

We illustrate the effectiveness of our feature vectors in the following manner. (1) Based on two well-known protein domain classification databases, CATH [17] and SCOP [18], we compare the accuracy of prediction using our moment vector versus other representations. We show that we achieve a much higher accuracy at all levels. (2) Using our moment vectors, we construct a 3D domain structure universe. We are able to show and visualize a clear and consistent distribution of domains in this universe. (3) We cluster the domains according to our moment vectors and demonstrate a relationship between structural variation and functional diversity.

## Methods

### Distance Matrix (DM)

**Figure 1:**
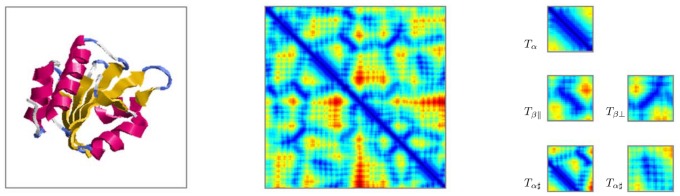
Representation of domain structure by its distance matrix, near contacts and their local image patterns. From left to right, SSE topology, distance matrix image and local patterns of SSEs and motifs are shown respectively. The example domain (d2c0ga2) selected from SCOP database belongs to c.47.1.7 category. 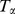 shows a deep blue strip near and parallel to main diagonal of DM, 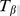 and 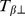 are deep blue slant strips parallel and perpendicular to main diagonal respectively, and 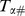 exhibits texture patches comprising horizontal and vertical blue strips. Entries of distance matrix are colored from blue to red according to their values from small to large.

Given a protein domain, we represent the distances between any two residues (using their 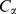 atoms as representatives) in a 2D matrix. An example is shown in Fig. 1. Since this distance matrix is symmetric, we only need to focus on the upper triangular part. To identify the protein secondary structure elements (SSE), we mainly look at the regions in which the residues are close together, i.e., the regions in the DM with deep blue colors (the deeper the color is, the closer the residues are). The reason is that in these structures, residues are bounded by strong hydrogen bonds in alpha helix and beta sheets or non-hydrogen bonds (such as ionic bonds, disulfide bonds, van der Waals/hydrophobic interactions that exist between helix and other SSEs, e.g. helix-sheet interaction in 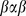 motif, 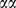 hairpin or closed 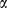 helix-coil part). These residues tend to be closer. In our approach, we do not need any input about what and where SSEs are in a given protein domain.

1. Alpha helix:

In a typical alpha helix, each spiral loop takes about 3–4 residues(3.6), say the 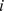-th residue to (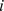
+3)-th residue, with the (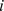
+3)-th residue closest to the 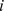-th residue and the (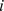
+4)-th residue wrapped overshooting the 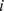-th residue. Since these 4 residues are very close together, thus in terms of the DM, we should see a deep blue strip with width of 3–4 residues near the diagonal (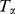 in Fig. 1). Furthermore, based on the findings in [19], the pairwise distances of these 4 residues exhibit a local minimum between the distance of the 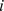-th and the (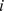
+3)-th residues. Thus, capturing the strip and identifying the local minimum within the strip, which is 3 residues from and parallel to the diagonal, should enable us to identify alpha helix.

**Figure 2:**
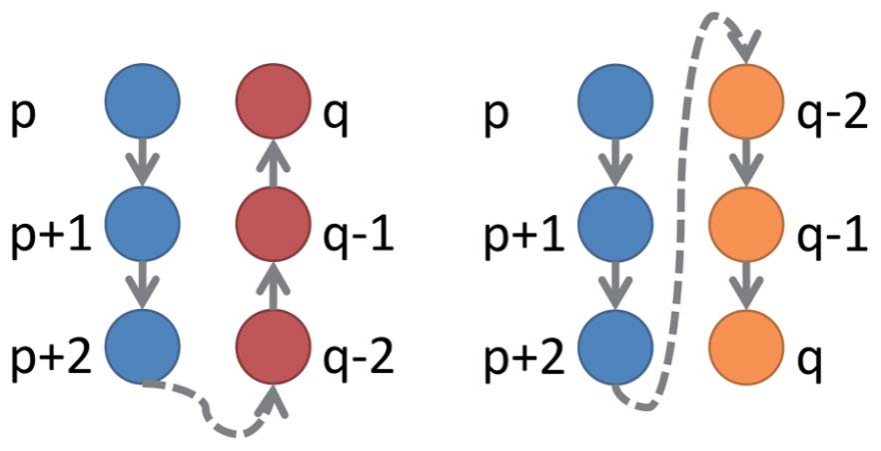
Structural diagram of anti-parallel and parallel *β* sheets. Each circle node represents an amino acid residue, and its position number in sequence is marked nearby. Arrows denote the direction of domain sequence from 5′ terminal to 3′ terminal. The dotted arrow linking node (p+2) and node (q-2) means that other residues exist between these two residues. Anti-parallel(blue and red nodes) and parallel(blue and orange nodes) 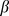 sheets are arranged side-by-side.

1. Beta sheets:

Two strands of a beta sheet are connected by hydrogen bonds. Depending on the orientations of these two strands (Fig. 2), the distances between these bounded residues (either{(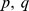); (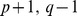); (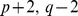)} in Fig. 2-A or {(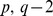); (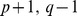); (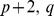)} in Fig. 2-B) are smaller compared to the distances between other nearby residues. Thus, in terms of DM, there again exists a blue strip either parallel (for parallel beta sheet) or perpendicular (for anti-parallel beta sheet) to the diagonal, but due to the extra residues in between the two sheets, this strip might not be near the diagonal (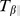 and 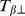 in Fig. 1). Identifying these strips parallel and perpendicular to the diagonal helps us to identify beta sheets.

1. Other structures:

There are other non-hydrogen bondings that occur between 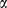 helix and other SSEs such as the helix-sheet part in 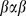 motif, 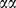 hairpin or the closed 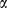 helix-coil part. Due to the periodic structure of alpha helix, we found that these bondings correspond to local minima in DM. For example, residue 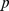 of a sheet is close to residues 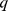, 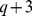 or 4, 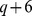 or 7 or 8, 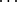 of a helix, similarly for residues 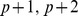 of the sheet, which are also close to the corresponding residues of the helix. Thus, there exists a narrow band of short blue horizontal or vertical strips of 3–4 residues apart (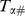 in Fig. 1). When two helices interact, both horizontal and vertical strips exist, cross each other and form textural patterns comprising ‘+’-shaped units.

### Gabor Filters and Binarization

Even though the above features are represented by relatively low values (since they are closer) in the DM, simple threshold filtering [10] cannot easily identify and separate these SSEs. We used the Gabor filter [20] to identify these SSEs from the images. Since simple cells in the visual cortex of mammalian brains can be modeled by Gabor functions, people tend to utilize Gabor functions to analyze image with the way similar to the perception in human visual system [20]. For example, human perceives static shapes by accumulating the information (orientations, boundaries or patterns, etc.) around them to enlarge contrast between shapes and their neighbors. Similarly, 2-dimensional Gabor function can enhance the contrast between local extrema (minima or maxima) and their neighboring entries in a matrix or an image by an accumulating operation, such as convolution. Gabor functions have been found to be particularly appropriate for the representation and discrimination of lines(local extrema), edges (local extrema) and texture (a spatial pattern of line or curve combination) [20].

**Figure 3:**
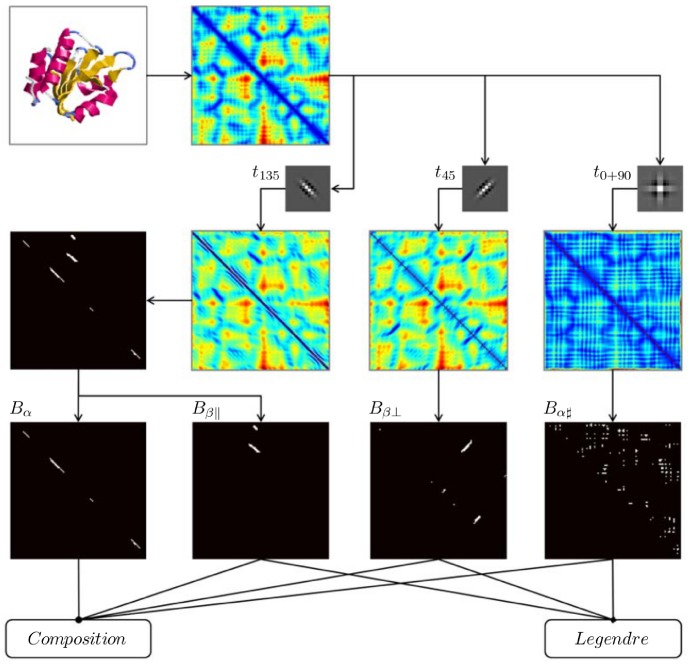
The work-flow of generating feature vector from domain structure(*c_α_*-atom coordinates). First, the distance matrix of domain structure (d2c0ga2, c.47.1.7) is calculated. Then, its distance matrix is passed concurrently through three Gabor templates(small patches tagged by 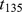, 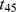 and 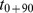 respectively). After that, four binary contact matrices 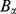, 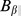, 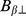 and 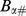 are output by filtering. Last, composition and Legendre moments are calculated to form the feature vector.

According to Fig. 1, in order to extract these SSE patterns from the images, we designed three Gabor templates: one for alpha helix and parallel beta sheet; one for anti-parallel beta sheet; and one for other structures. The match between Gabor templates and patterns in image is highly specific. The technical details are given below. Gabor functions 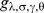 in four orientations *θ*  =  0°,90°,45°,135° were selected, where wavelength 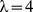, the spatial aspect ratio of major axis to minor 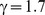 and the length of major axis in the Gaussian elliptical envelop 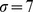. Then, three templates 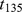, 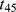 and 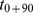 were set up, corresponding to Gabor functions in orientation 135°,45°,0°+90°, for filtering, in which the sizes were all 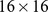, where *t*_135_ = g_135°_ was used for 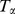 and 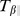, 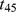 for 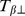 and 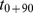 for 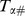 (see the templates in Fig. 3).

We performed the Gabor filtering by the two-dimensional convolution of DM and three templates independently to obtain three groups of patterns of low values. Three binary contact matrix (BCM) were then constructed by assigning 1 to those elements whose convoluted values in the convoluted DM are less than or equal to zero and assigning 0 to the remaining positive values. The binary slant line 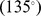 patterns of 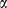-helices and parallel 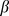-sheets in the BCM derived from template 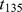 were further separated to form two BCMs according to their closeness to the main diagonal (the slant line patterns of parallel 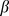-sheets are further away from the diagonal than those of the 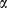-helix). Finally, we have four BCMs marked as 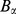, 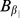, 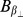 and 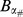 respectively (Fig. 3). The count and type of 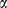 helices and 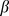-sheets from the BCMs are highly consistent with domain structure. More examples are shown in Fig. S1.

### Structural Analysis by Higher-Order Moments

These four BCMs can be treated as four binary images containing different information of the SSEs. Each set of the adjacent 1’s (edges or edge patterns) in each BCM represents one type of SSE or some SSEs’ pairwise interactions. To capture the characteristics of these four BCMs, we define the first set of moments as follow and refer to them as Composition Moments:
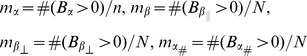
(1)where 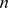 is the sequence length of the domain(domain size), 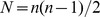 is the area of right-top part of BCM, and 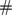 denotes the count of elements that satisfy the specific condition in BCM. In terms of residue-residue contact, the moments 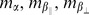 and 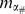 are the average count of four types of near contacts occurring in the protein structure respectively. In particular, 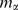 is the composition of helix in a domain structure, 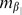 and 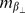 are the composition of parallel and anti-parallel sheets, and 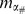 is the composition of hydrophobic contacts.

It has been shown that any image, in principle, can be recovered by a sufficient number of image moments [21]. That is to say, image moments contains all information in image. Similar to orthogonal polynomials, orthogonal moments, e.g., Legendre moments, can be used to capture the image information. Legendre moments are derived from and equivalent to geometric moments but with considerably computational benefits, such as a stable and fast numerical implementation, the avoidance of the loss of precision caused by overflow or underflow, and a higher robustness to random noise [21]. According to geometric moments, for a given shape, the 0 order and the 1-order moments are its area and mass center respectively, the 2-order moments are its Newtons inertia moments which define shapes resistance to change of 3 axes of rotation, the 3-order moments measure the degree of asymmetry of shape (skewness), and the 4-order moments measure the degree of locally extreme irregularity in shape (kurtosis). Five-order or higher-order moments are also used in many application though they are still not easy to explain in a conceptual way [21].

Analogous to Fourier transform, the relationship between different orders of moments and BCM is similar to the relationship between different frequencies and the signal, where high frequencies represent higher-order information of the signal while low frequencies give the signal’s outline. The lower-order Legendre moments depict the outlines of slant lines or maze-like patterns, and the higher-order Legendre moments capture their higher-order information. In the context of BCM, for example, the moments of layout of 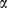-helices has the following meanings: (1) 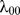 shows how many residues belonging to 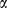-helix, (2) the 1-order moments show where the center of 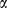-helices in domain is according to the sequence positions, (3) the 2-order moments define how helices span domain according to the sequence positions, (4) the 3-order moments measure the degree of asymmetry of helix distribution along the sequence and (5) the 4-order moment denote the deviating degree of the extreme helix from other helices according to the sequence positions. This is usually caused by the insertion of other SSEs between the extreme helix and other helices.

For anti-parallel 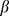-sheets of a domain in BCM, the moments of their layout can be generally explained as follows. (1) The 0-order moment shows how many residues involving in anti-parallel 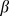-sheets. (2) The 1-order moments show the center of anti-parallel 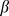-sheets in BCM according to participating residue pairs. (3) The 2-order moments measure how anti-parallel 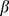-sheets span BCM. For example, two domains contain the same motif of meanders which have the same count of residue pairs in anti-parallel 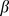-sheets but they may still have different 2-order moments if the intervals between their member strands in sheets differ. (4) The 3-order moments measure the layout asymmetry of anti-parallel 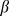-sheets in BCM. For example, the difference of meander and Greek-key motifs in BCM can be represented by the 3-order moments because meander motif can show a symmetric layout in BCM but Greek-key motif surely shows an asymmetric layout along the diagonal of BCM. (5) The 4-order moments measure how deviating an outlier sheet is from other sheets in BCM and have large values when the deviation is large. The meaning of moments of other two kinds of near contacts can be explained in similar way. Therefore, the Legendre moments represent a gradual approximation and detailed characterization of domain structure through the slant lines or maze-like patterns, which correspond to the SSE, and their interactions. In addition, moments can be treated as pattern features in image and vision analysis in many applications because of the invariant properties, such as scaling and translation and the insensitivity of image noises and the avoidance of disadvantages on structural alignments [21].

The Legendre moments 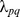 of order 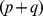 are defined as
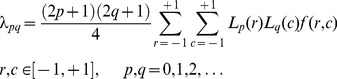
(2)where 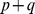 is the order of moment, 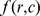 stands for an image intensity function at the normalized row value 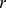 and column value 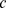, and 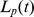 is the Legendre polynomial of degree 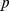.

In our study, only the first five degrees of the Legendre polynomials are considered,
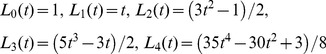
(3)


Thus only those moments 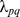 with the order 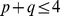 are used in experiments.

## Results

In this section, we shall demonstrate that moment features are superior in many aspects, such as protein domain classification, domain structure and function representation, when compared with other approaches.

### Classification Performance

We first illustrate the classification power of the image moments. Our composition moments and all additional Legendre moments 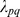 with order 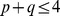 were used in the experiments. In Table S1, we study the increase in effectiveness of classification by using Legendre moments with different higher orders. The same datasets of CATH domains and the same training and testing schemes as those in the recent work [11] were adopted. We downloaded the top 500 H-level domains(denoted by CATH_32 with 98,110 domains) from CATH v3.2 and the new domains in CATH v3.3(denoted by CATH_33New with 14,437 domains). The classification scheme adopted a 3-fold cross-validation that two-thirds of CATH_32 were randomly sampled for training and the remaining one-third of CATH_32 and all domains of CATH_33New were used for testing respectively. This scheme was repeated three times and its final accuracy is the average of accuracies resulted in three trials.

We compared the classification performance of the image moments with the algebra-topological description, a most recent approach proposed by [11]. The classification accuracy is defined as the percentage of correctly classified domains according to the classification in CATH. To have a fair comparison, we also used the same classifier (Random Forest, RF) as that proposed in their paper. In addition, we also tried Support Vector Machines (SVM) using our moments. The results in Table 1 show that moment features give better classification performance than the algebra-topological description for all classification levels. In particular, for the lowest classification level(H), the accuracies of using moment feature are more than 20% higher than others in both CATH_32 and CATH_33New testing datasets. This demonstrates that moment features can be more effective in capturing the structural characteristics of domains. As a by-product, we also see that SVM gives a slightly better result than RF. Because the datasets used in a recent work [11] were collected several years ago, we also downloaded the latest version (v3.4) of top 500 H-levels in CATH to validate the classification performance of our proposed features by the 3-fold cross-validation. The classification of total 129,739 domains is consistent with CATH according to the accuracies of 99.8412%, 99.4441%, 99.3763% and 99.1916% in C, A, T and H levels respectively.

**Table 1:** Comparison of methods on the top 500 superfamilies of CATH.

Method	Classifier*	C† (%)	A† (%)	T† (%)	H† (%)
Ref. [11]	RF	96.1/92.8†	84.6/74.1	78.4/63.0	74.9/55.3
Moment	RF	99.6291/96.5921	98.9437/87.6913	98.7644/79.8088	98.3783/72.2882
Moment	SVM	99.8022/95.9825	99.512/89.2429	99.3914/82.4686	99.1258/77.2321

RF:Radom Forest, SVM:Support Vector Machines.

The four levels, C, A, T and H contain 4, 33, 328 and 500 types respectively.

The value pair separated by “/” in each cell means the accuracies of classification on CATH_32 and CATH_33New.

We also evaluated the moment features using another popular classification database SCOP(v 1.75). Again, the top 500 superfamilies (totally 109, 533 domains, denoted as S_H500) were selected for evaluation. Following a similar training scheme as above, we found that the results of classification are also consistent with those of SCOP in all levels (Table 2).

**Table 2:** Agreement with SCOP classification using moment.

Classifier*	Class† (%)	Fold† (%)	Superfamily† (%)
RF	99.5131	98.5392	98.1515
SVM	99.7870	99.3166	99.2058

RF:Radom Forest, SVM:Support Vector Machines.

Class, Fold and Superfamily levels contain 4, 352 and 500 types respectively.

We found that Legendre moments are powerful enough to distinguish folds within a class (see Fig. S2) and superfamilies within a fold (see Fig. S3). This explains why moment features can achieve much higher classification accuracies even for more fine-grained classification levels. Note that in both evaluations of CATH and SCOP, SVM seems to perform better, so we used SVM in our experiments. In the rest of the paper, we focus on SCOP classification because SCOP treats 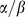 and 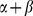 domains separately whereas CATH merges them into mixed 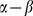 class.

### Domain Structure Universe

In this section and the next section, we demonstrate another two more useful applications of moment features. One approach to investigate the relationship between protein structure and function is to represent protein structure in a high dimensional space, e.g. the three-dimensional maps generated by dimension reduction, such as multidimensional scaling (MDS) [22]–[24], singular vector composition (SVD) [10] and principal component analysis (PCA) [3]. Such 3-D maps are not only good for human visualization, they are capable of (i)representing the distribution of domain size (peptide length), types of secondary structural elements in class level [10], [11], [22], (ii) inferring protein functions [23], tracing the “common structural ancestor” [24] and (iii) analyzing the distribution of functional diversity [3].

Though MDS, PCA and SVD (related to PCA strongly) give a good 3-D visualization to domain structure maps, it is difficult to visualize the spatial relationship between small-size and middle-size domains as they always cluster closely together in such space. This problem becomes more eminent as most domains (84.51%) belong to these categories of sizes smaller than 300 peptides. The sizes of domains have been shown to follow approximately the power-law distribution (Fig. S4). In addition, these methods of dimension reduction cannot work well on non-linear data [25]. Kernel Principal Component Analysis(KPCA) has been designed to address dimension reduction for non-linear data [25], and the dimension reduction through KPCA provides an excellent data visualization in bioinformatics, such as gene expression [26], [27] and protein phylogenetic profile [28]. Since both KPCA and SVM share the same kernel trick which is the core of all kernel methods, we used KPCA to visualize the feature space while keeping the classification consistency property of SVM.

To illustrate that the moment features can also perform well in this application, we chose 3,000 domains from non-redundant subset of S_H500 (with less than 40% sequence similarity). The feature vector of each domain is represented as a point in the 49-dimensional space. We applied KPCA to map these points non-linearly onto a reproducing kernel Hilbert space in which the original linear operations of PCA were performed [25]. The first three principal components of newly mapped points were taken to form domain structure universe.

**Figure 4:**
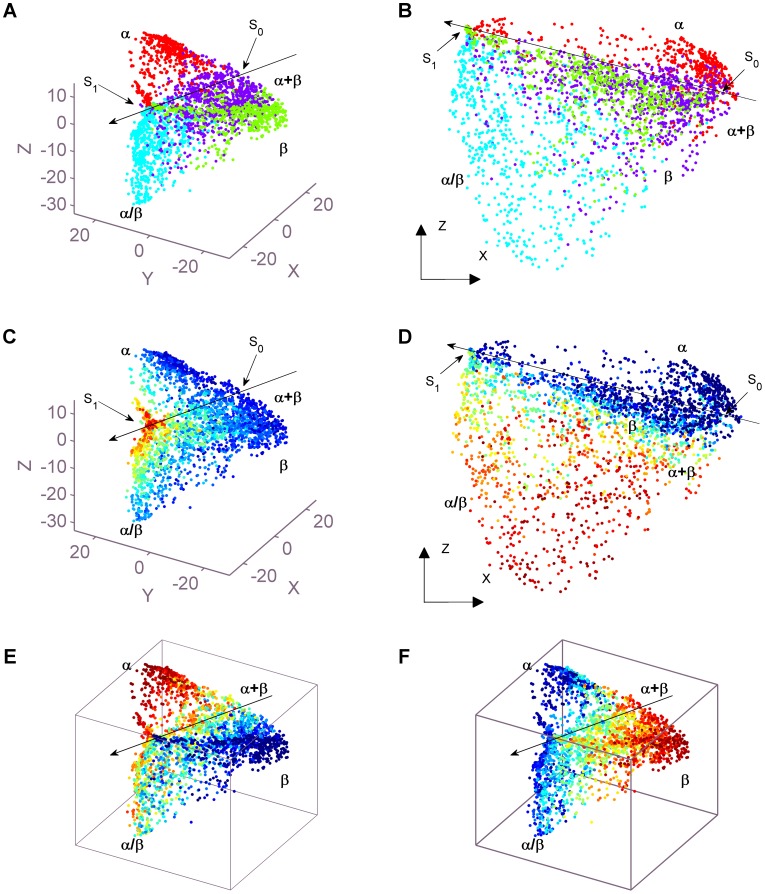
Maps of domain structure universe. (A)(B)Class map in two viewpoints. All-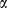 (red), all-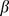 (green) and 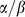 (cyan) domains are clustered into three distinct zones which appear quite separately in three planar region respectively. In particular, 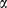 region and 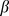 region are sickle-shaped but nearly mirror-symmetric, while 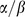 region is wing-shaped. Moreover, the extensions of three planar regions intersect approximately at an axis with an included angle of about 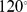 from each other. On the other hand, 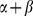 (purple) domains appear between aforementioned regions and mix slightly with them. In (A)(B), red for all-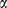, green for all-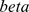, cyan for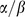, purple for 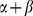. X, Y and Z denote the first three principal components respectively. (C)The distribution of domain sizes. This map is shown in the same viewpoint of (A). Domains are gradually rendered from blue to red corresponding to their sizes from small to large. According to Class map, two joint points, 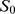 and 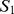 between three regions are found on the intersecting axis(denoted as a long arrow) of planar regions. 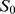 is only shared by the sickle points of 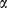 and 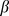 sickle-shaped regions while 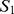 is the meeting point of all regions. Domains close to 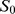 are small. When moving away from 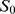 to 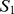 along with 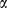, 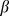 and 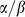 regions respectively, the sizes of domains gradually increase. In other words, the intersecting axis of planar regions shows the significant trend of domain sizes from small to large. (D), (E) and (F) are the composition maps of 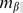, 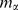 and 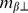 respectively. In (D)-(F), similar color schemes are adopted to label domains from blue to red according to their composition feature values from small to large. (D) is shown in the same viewpoint of (B) while (E) and (F) are shown in the same viewpoint of (A). (D) shows that domains in 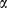 and 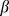 regions have small values of 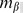, domains deviating from 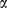 or 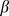 regions and domains in 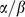 regions have large values of 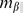, and the larger the value is, the farther the domain deviates downward from 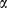 or 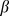 regions to the edge of 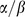 regions. (E) shows that domains in 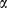 region and 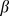 regions have large values and small values of 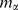 respectively. When moving from the leftmost point of 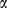 region to the intersecting axis and to the rightmost point of 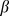 region, the 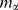 values domains gradually decrease. Domains in 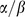 region also follow the trend and they are just located on the intersecting axis according to the moving direction so as that they have median values of 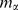. (F) shows that domains in 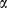 region and 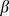 regions have small values and large values of 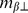 respectively. When moving in the aforementioned direction, the 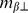 values domains gradually increase. Again, domains in 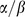 region follow the trend.

To represent the universe, diverse maps of the selected dataset are shown in Fig. 4 and their more perspectives of different viewpoints are shown in Fig. S5. Three types of maps are rendered by different color schemes which are corresponding to their diverse attributes. (1) The first one is Class map shown in both Fig. 4-A and Fig. 4-B in 2 different viewpoints. All-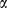 (red), all-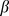 (green) and 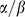 (cyan) domains are clustered into three distinct zones which appear quite separately in three planar regions respectively. In particular, both 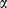 region and 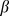 region are sickle-shaped but nearly mirror-symmetric to each other (Fig. S5-A provides a better perspective.), while 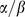 region is wing-shaped. Moreover, the extensions of three planar regions intersect roughly at an axis(denoted as a long arrow) with an included angle of about 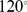 from each other. On the other hand, 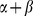 (purple) domains appear between aforementioned regions and mix slightly with them. Some 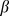 domains, such as 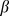-helix folds, deviate from 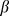 planar region because of more parallel 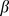-sheets occurring in all-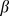 domains (Fig. S5-B). (2) The map in Fig. 4-C shows the distribution of domain sizes in the same viewpoint of Fig. 4-A. Domains are gradually rendered from blue to red corresponding to their sizes from small to large. According to Class map, two joint points, 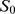 and 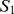, between three regions are found on the intersecting axis of planar regions. 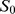 is only shared by the sickle points of 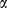 and 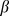 sickle-shaped regions while 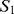 is the meeting point of all regions. Domains close to 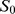 are small. When moving away from 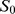 to 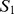 along 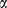, 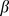 and 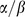 regions respectively, the sizes of domains gradually increase. In other words, the intersecting axis of planar regions shows a significant trend of domain sizes from small(blue) to large(red). (3) Fig. 4-D, E, F and Fig. S5-R represent four composition maps of near contacts for domains respectively. Fig. 4-D is shown in the same viewpoint of Fig. 4-B while Fig. 4-E and Fig. 4-F are shown in the same viewpoint of Fig. 4-A. These composition maps are rendered by similar color schemes which label domains from blue to red according to their composition feature values from small to large. Fig. 4-D shows that domains in 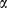 and 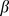 regions have small values of 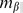, domains deviating from 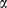 or 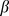 regions and domains in 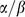 regions have large values of 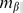, and the larger the value is, the farther the domain deviates downward from 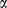 or 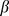 regions to the edge of 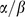 regions(see also Fig. S5-H). Fig. 4-E shows that domains in 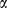 region and 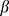 regions have large values and small values of 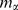 respectively. When moving from the leftmost point of 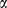 region to the intersecting axis and to the rightmost point of 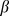 region, the 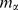 values domains gradually decrease (see also Fig. S5-I from top to bottom). Domains in 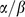 region also follow the trend and they are just located on the intersecting axis according to the moving direction so as that they have median values of 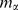 (see also Fig. S5-J). Fig. 4-F shows that domains in 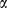 region and 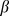 regions have small values and large values of 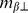 respectively. When moving in the aforementioned direction, the 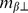 values domains gradually increase(see also Fig. S5-L from top to bottom). Again, domains in 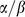 region follow the trend(see also Fig. S5-M). Fig. S5-R shows the trend of 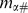 and a similar change as in Fig. 4-E, therefore it validates that most non-hydrogen bondings occur between 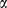-helix and other SSE. Similar class maps and composition maps of the whole S_H500 and CATH_32 datasets (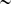
100,000 domains) are also shown in Fig. S6 and Fig. S7 respectively.

Overall speaking, the architecture of our domain structure universe is consistent with those of former works [10], [22] in terms of the following aspects. (1) Most of 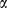, 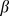 and 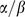 domains are grouped into three separated Class clusters of which each shows roughly a plane(called Class plane), and 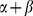 domains occur in their joints. (2) The size of domains increases gradually when moving away from the small-size point to the small-large point along with 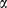, 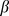 and 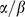 planes respectively. Moreover, fewer 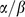 domains occur at the small-size region than other classes of domains. (3) The type of SSE including 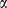-helix and 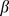-strand, and the parallelism of 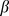-sheets between 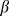 and 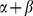 domains are also strongly related to corresponding Class planes respectively [10]. (4) Some of 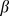 domains deviate greatly from 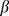 plane due to their types of 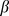-roll or 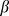-helix folds which contain more parallel 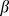 sheets than anti-parallel 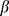 sheets [22]. (5) Additionally, the extension of our 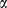, 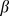 and 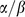 planes intersecting at a specific angle is similar to the universe in [10], but is slightly different from the universe in [22] which suggests that 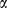 and 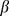 domains are coplanar and 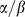 plane is perpendicular to the 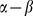 plane. On the other hand, our universe has other advantages. First, a better visualization of domain distribution is provided for small- or middle-size domains rather than grouping them together (what other works did). This is important as most domains are less than 300 in size. Secondly, a quantitative visualization of near residue-residue contacts, including hydrogen bonds and non-hydrogen interaction, is provided by the corresponding composition features.

### One Utility of Domain Structure Universe: Function Diversity of Superfamily

A set of domains in a superfamily has a common functional ancestor [29]. Therefore, the analysis of structural domain superfamilies provides an important insight for the exploration of the evolution of protein structure and function. Although domain undergoes structural changes during evolution, the structural diversity of domains in a superfamily is caused by their extensive structure embellishments and not by the common core which is shared by all members in the superfamily [2]. More importantly, structural diversity in a superfamily plays a crucial role in functional variations [2], [30]. An evolutionary explanation can be found in [31].

The variations of domain combinations can make significant contribution to the evolution of organismal function [29]. The creation of new proteins is predominantly caused by frequent rearrangements of existing domain combinations, including duplication and permutation, rather than *de novo* creation [31]–[33]. Consequently, the functional diversity of superfamily is mainly caused by both the structural variation of domains [2] and the combination arrangement of domains [34]. Here we attempt to make a link between structural variation and combination diversity of domain within a superfamily. We measure structural variation by the number of clusters in our structural space of domains. We believe that our moment vectors can provide enough information for grouping domains with similar structures together, thus the number of clusters could reflect the number of structural variations(that is also related to the diversity of functions) among the domains. We denote the number of clusters for each superfamily as 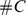, which is determined automatically by Mean Shift clustering method [35], [36] (of which the whole procedure is described in Section S1).

**Figure 5:**
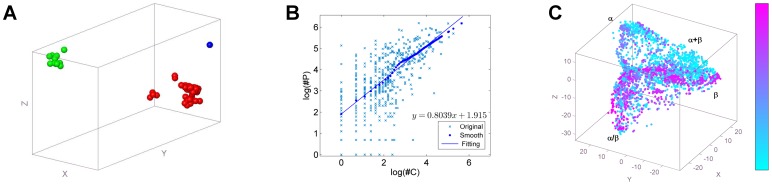
The relationships between structural variation and domain combination in superfamily. (A)The domain clusters of superfamily a.60.9. The superfamily shows three clusters in the proposed structural space. Red(48 domains), green(12 domains) and blue(1 domain) clusters represent exactly three types of tyrosine recombinases: Recombinase XerD, Flp recombinase and Cre recombinase respectively. (B)The relationship between the count of clusters (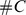) and the count of non-repeated partners 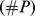. The logarithmic values of 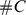 and 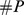 in selected superfamilies, their smoothed values and the fitting line are drawn together to illustrate the significant linear relationship between 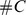 and 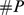 in logarithmic coordinates system. (C)Non-repeated partners map for superfamilies. Domains are labelled from cyan to purple according to 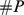 values of their corresponding superfamilies from small to large. Most of superfamilies holding large 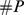 appear in 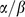 plane, and some in 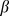 plane, especially near the edge of 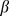 plane.

Using 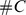 as a measure of structural variation across a superfamily has also been used in previous work [37]. In order to validate such measurement, another independent measure of structural variation is calculated by a pairwise structural alignment algorithm, called jFATCAT (freely available in http://www.rcsb.org/pdb/workbench/workbench.do). We selected two superfamilies, a.60.9 and a.69.1, from SCOP (V1.75) and investigated the alignment scores between domains in each superfamily respectively. The higher the alignment score is, the less structural variations two domains have. The detailed scores are listed in Score S1. In the proposed structural space, the superfamily a.60.9 (SCOP Name: *lambda integrase-like, N-terminal domain*) shows 3 clusters which contain 48, 12 and 1 domains and are rendered by red, green and blue respectively (see Fig. 5-A). The ranges ([minima, maxima]) of alignment scores within each cluster and between any cluster pair are calculated. In details, the range of alignment scores within the red cluster is [292.48, 335.52], while the score range between red and green clusters is [106.41, 128.05] and that between red and blue clusters is [153.82, 177.25]. And the score range within green cluster is [313.57, 359.92] while the score range between green and blue clusters is [114.83, 123.53]. Obviously, the scores are higher within each cluster, the scores are lower between domains in different clusters, and there is even no overlap between score ranges of domains within-cluster and between-clusters. This provides an additional independent evidence showing that moment features are useful to classify structural difference of domains. More importantly, three clusters just represent three different types of tyrosine recombinases (*Flp recombinase, Cre recombinase and Recombinase XerD*) which share conserved DNA binding mechanism in recombination reaction, but also show apparent mechanistic and regulatory differences [38]. Another superfamily a.69.1(SCOP Name: *C-terminal domain of alpha and beta subunits of F1 ATP synthase*) also shows similar results of structural comparison. It has 2 spatial clusters in structural space, each of which is composed of 47 domains. One cluster denotes *alpha subunit* of *F1 ATP synthase* and another denotes its *beta subunit*. Three *beta subunits* in *F1* component are the ATP-ADP binding sites whereas *alpha subunits* are not sites but just support the *F1 ATP synthases* structure, even though they are placed together with the alternating arrangement in *F1 ATP synthase* [39]. Consequently, the above results demonstrates that structural variations of domains in a superfamily strongly related to the diversity of their functions according to the annotations in SCOP, and also illustrate that 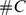 can capture the number of diverse functions, despite the fact that the domains can still have common conserved functional features, in a superfamily.

Combination diversity is investigated by SUPERFAMILY which provides a domain-based gene ontology at the superfamily level [40]. A protein consists of one or more domains and a multi-domain protein generally show a specific combination of domains according to domain counts and superfamily types [40]. Here, unique superfamily arrangement of domains is defined as the unique sequential combination of superfamily labels of member domains in a protein and a unique arrangement usually can be usually shared by multiple proteins. For example, considering 2 proteins, one contains sequentially three domains which belong to 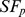, 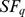 and 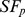 superfamilies respectively, so it holds the domain arrangement 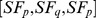. If another protein has the same arrangement in terms of superfamily, then only one unique arrangement occurs between two proteins. If not, such as 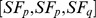, two unique arrangements are counted. Here, 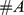 is defined as the number of unique superfamily arrangements for a given superfamily. Moreover, the set of non-repeated 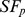-exclusive superfamilies in the unique arrangement 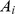 including superfamily 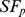 is called 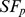
’s partners in 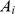. For all unique arrangements 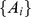 including 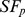, a non-repeated set of its partners in 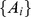 can be obtained. For example, given three unique arrangements 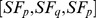, 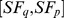 and 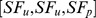. The unique set of 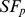
’s partners is 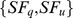, while 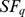
’s unique partner and 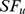
’s unique partner are only 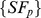. Here, 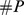 is defined as the number of non-repeated partner superfamilies in all unique arrangements for a specific superfamily. These two counts, 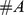 and 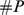, are able to approximate the function diversity of the superfamily in a large extent [40]. As the information in SUPERFAMILY is not complete, the two counts are missing in some superfamilies, such as, *viral protein domain* superfamily(b.19.1). In total, 475 superfamilies with both non-zero values of 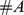 and 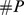 from the dataset S_H500 are studied. The Spearmans correlation coefficients of 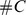 to 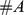 and 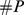 are 0.6234 and 0.6785 respectively with 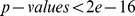, with larger Spearmans correlation coefficients 0.6763 and 0.7238(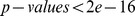) to 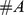 and 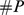 respectively in the case of bigger superfamilies (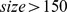). Therefore, the count of clusters (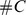) shows significant correlation with the count of non-repeated partners 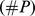 and the count of unique arrangements 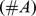.

To investigate the relations between 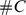, 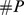 and 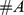 values, we applied a robust smoothing algorithm [41] to eliminate the noise in 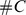, 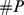 and 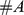, and then provided a fitting. By considering potential power-law-distribution, we used natural logarithm of values in 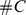, 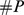 and 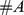 instead of their original values. Smoothed data shows a linear relationship between 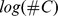 and 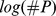 (Fig. 5-B) or 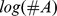 (Fig. S8), i.e. the relationships between 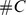 and 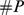 or 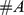 follow the power-law-distribution. We studied the smoothed 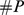 of each superfamily in domain structural space by rendering the points according to the smoothed values of 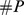 (Fig. 5-C). Most of superfamilies with big 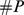 appear in 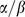 plane, and some in 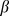 plane, especially in the edge of 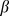 plane. This result may be explained by the conformational stability of proteins, with the 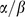 folds most stable, followed by the all-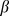 folds [42]. The more stable the protein is, the higher degree of mutation it can tolerate [31]. Accordingly, the superfamilies including stable proteins have more chances to produce diverse embellishments in evolution.

From the viewpoint of structural space, the more clusters a superfamily has, the bigger structural variation it has, the more diverse its embellishments are, and the more variant functions occur within superfamily [2], [30]. From the viewpoint of evolution, the larger 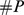 or 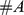 of a superfamily are, the higher diversity of function the superfamily has. Our moment feature representation links up these two aforementioned views and supports the hypothesis that the evolution might be more disposed for selecting domains from a superfamily with a higher structural variation in order to produce new proteins with domain combination [31]–[33].

## Discussion

Based on the image patterns of the hydrogen-bond contacts of 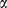-helix and 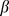-sheet and other hydrophobic contacts in distance matrix, we decompose the distance matrix image into basic binary images representing elementary SSEs and motifs of domain structures. By further deriving image moments from these basic images, we propose a moment feature vector to capture the structural characteristics of a protein domain. This feature vector was demonstrated to be useful in improving the domain classification accuracy, and provided a clear domain structure universe for the study of the distribution of domains. The findings of the distributions (e.g. length) are consistent with [22] and [10].

The same structure universe also demonstrates a positive relationship between the degree of structural variation (approximated by the number of clusters of the superfamily in the universe) and functional diversity (approximated by the number of non-repeated partners and the number of unique arrangements of the superfamily). Moreover, in the structure universe, the partner map of domain combination shows a significant distribution of superfamily combination diversity.

Our work can be explored for other applications. A popular utility is to query some newly determined structures with unknown functions. Mapping it into a structure universe and comparing to other domains will give its structural classification. The feature vector with existing retrieval techniques in computer vision provides an efficient search of structural similarity. Furthermore, diverse moments can be expected to contribute to model domain structure mathematically, and can be used for structure prediction. Finally, the binary contact matrix can be used for other applications, for example, automatic detection of the common structural core within a superfamily and automatic multi-domain decomposition.

## Supporting Information

Figure S1
**Comparison of different classes of domains.** The structures, distance matrices and four binary contact matrices including 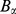, 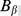, 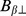 and 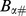 are listed from left to right. The names of four domains and their lineage of SCOP classification are d1fnna1(a.4.5.11), d1beba_(b.60.1.1), d2c0ga2(c.47.1.7), and d1tdja2(d.58.18.2) from the top down. Only the upper triangular part of each BCM is used because of its symmetry.(TIFF)

Figure S2
**The classification of different folds within a class.** Three folds, a.51 (Cytochrome c oxidase subunit h, 4 helices, irregular array, disulfide-linked,46 domains), a.52 (Bifunctional inhibitor/lipid-transfer protein/seed storage 2S albumin, 4 helices, folded leaf, right-handed superhelix, 47 domains) and a.56(CO dehydrogenase ISP C-domain like, 4 helices, bundle, 45 domains) are investigated. For two given folds, one of moments of 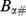 can separate them according to the histogram of moment values. The height of each bar is the count of domains within a specific range of moment values.(TIFF)

Figure S3
**The classification of different superfamilies within a fold.** Three superfamilies, a.4.7(Ribosomal protein L11, C-terminal domain, 105 domains), a.4.8(Ribosomal protein S18, 72 domains, and a.4.12 (TrpR-like, contains an extra shared helix after the HTH motif, 42 domains) are investigated. For two given superfamilies, one of moments of 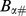 can separate them according to the histogram of moment values. The height of each bar is the count of domains within a specific range of moment values.(TIFF)

Figure S4 **The distribution of domain sequence length.** The fitting function is a power law function and the fitting performance is indicated by R-square = 0.9917 and RMSE = 9.93.
(TIFF)

Figure S5
**Map of domain structure universe with more perspectives.** Class map is shown in X-Y view (A) and Y-Z view(B). Size map is shown in X-Y view(C), Y-Z view(D), and X-Z view(E). Composition map of 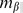 is shown in X-Y view(F), Y-Z view(G), and a 3-D view(H). Composition map of 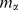 is shown in X-Y view(I), Y-Z view(J), and X-Z view(K). Composition map of 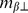 is shown in X-Y view(L), Y-Z view(M), and X-Z view(N). Composition map of 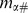 is shown in X-Y view(O), Y-Z view(P), X-Z view(Q), and a 3-D view(R). The color schemes are the corresponding ones used in Fig. 4 and described in main text.(TIFF)

Figure S6
**The maps of domain structure universe of SCOP.** Totally, 109,533 domains are drawn in maps. (A) In the map of class, red for all-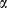, green for all-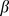, cyan for 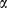/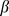,purple for 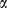
+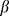. (B) Map of 500 Superfamilies. (C)–(F) Map of composition moments: 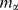, 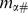, 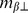 and 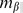, and the values of composition moments go incrementally from blue to red.(TIFF)

Figure S7
**The maps of domain structure universe of CATH.** Totally, 98,033 domains are drawn in maps. (A) In the map of class, red for mainly 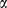, green for mainly 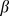, cyan for mixed 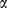-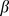,purple for small protein. (B) Map of 500 Superfamilies. (C)-(F) Map of composition moments: 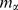, 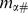, 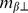 and 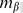, and the values of composition moments go incrementally from blue to red.(TIFF)

Figure S8
**The relationship between the count of clusters (#** ***C*** **) and the count of unique arrangements (#** ***A*** **).** The logarithmic values of 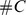 and 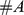 in selected superfamilies, their smoothed values and the fitting line are drawn together to illustrate the significant linear relationship between 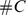 and 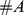 in logarithmic coordinates system.(TIFF)

Table S1 **Comparison of classifications on S_H500 with different feature groups.**
(PDF)

Section S1 **Mean shift clustering.**
(PDF)

Score S1 **Alignment Scores of Domains.**
(XLSX)
