# Supplementary figures and images for: Effective Moment Feature Vectors for Protein Domain Structures
Source: PLoS One. 2013 Dec 31;8(12):e83788. doi: 10.1371/journal.pone.0083788 (PMC3877117; doi:10.1371/journal.pone.0083788)

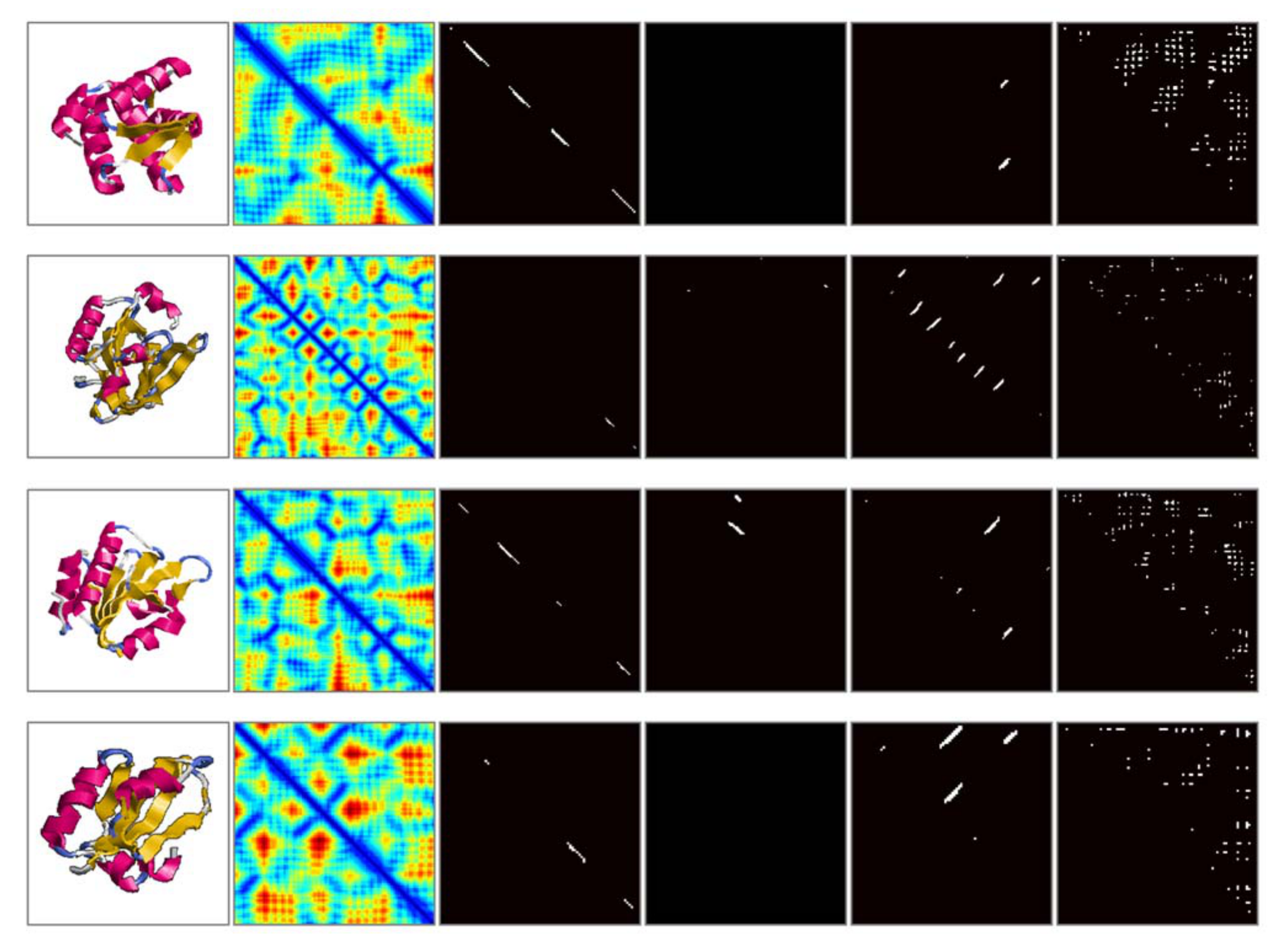

Supplement: Figure S1 — Comparison of different classes of domains. The structures, distance matrices and four binary contact matrices including , , and are listed from left to right. The names of four domains and their lineage of SCOP classification are d1fnna1(a.4.5.11), d1beba_(b.60.1.1), d2c0ga2(c.47.1.7), and d1tdja2(d.58.18.2) from the top down. Only the upper triangular part of each BCM is used because of its symmetry. (TIFF) [file pone.0083788.s001.tiff]

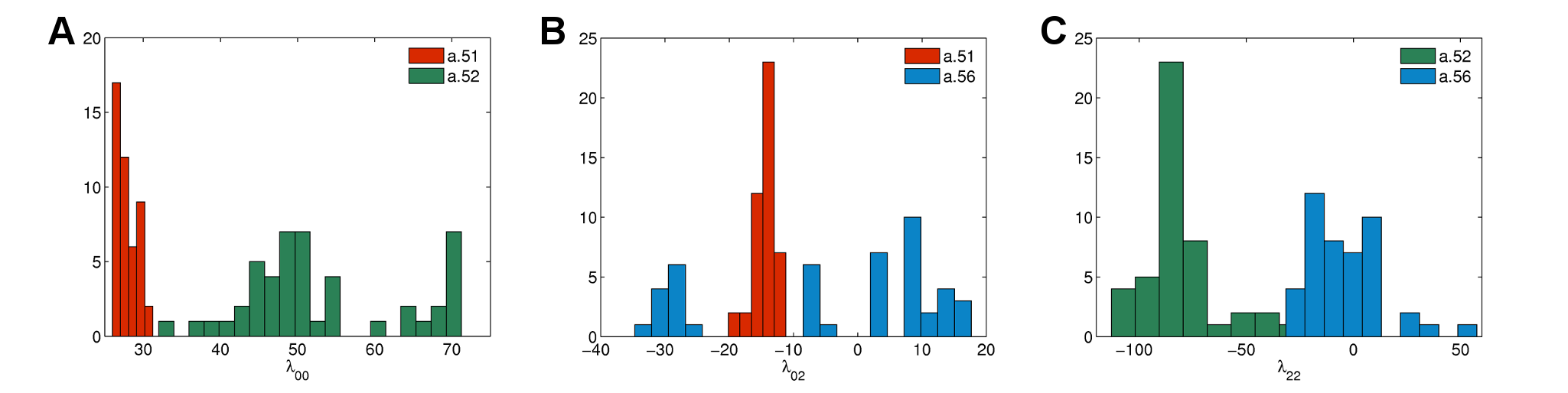

Supplement: Figure S2 — The classification of different folds within a class. Three folds, a.51 (Cytochrome c oxidase subunit h, 4 helices, irregular array, disulfide-linked,46 domains), a.52 (Bifunctional inhibitor/lipid-transfer protein/seed storage 2S albumin, 4 helices, folded leaf, right-handed superhelix, 47 domains) and a.56(CO dehydrogenase ISP C-domain like, 4 helices, bundle, 45 domains) are investigated. For two given folds, one of moments of can separate them according to the histogram of moment values. The height of each bar is the count of domains within a specific range of moment values. (TIFF) [file pone.0083788.s002.tiff]

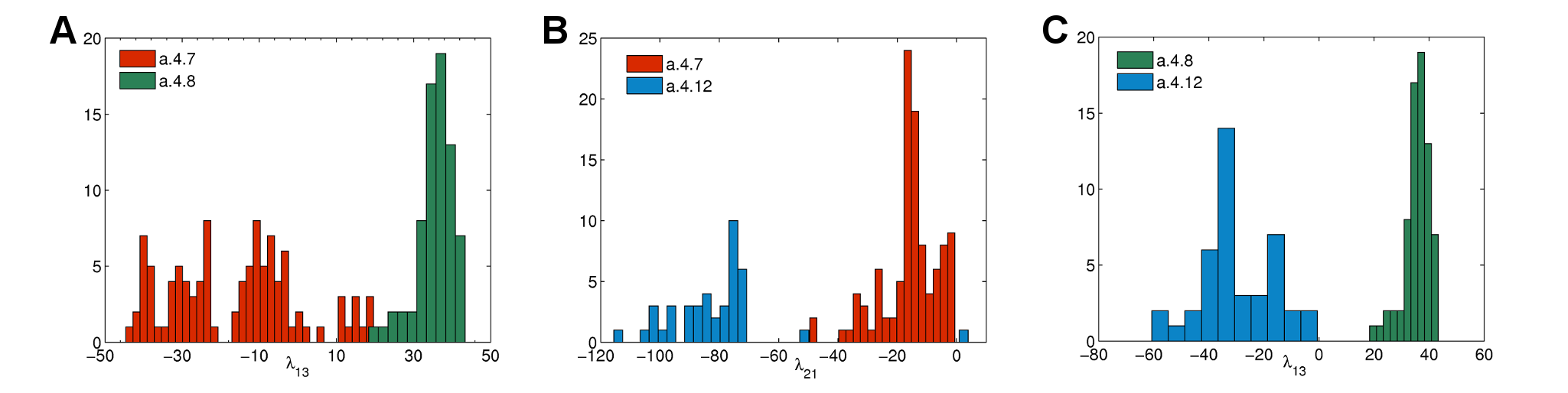

Supplement: Figure S3 — The classification of different superfamilies within a fold. Three superfamilies, a.4.7(Ribosomal protein L11, C-terminal domain, 105 domains), a.4.8(Ribosomal protein S18, 72 domains, and a.4.12 (TrpR-like, contains an extra shared helix after the HTH motif, 42 domains) are investigated. For two given superfamilies, one of moments of can separate them according to the histogram of moment values. The height of each bar is the count of domains within a specific range of moment values. (TIFF) [file pone.0083788.s003.tiff]

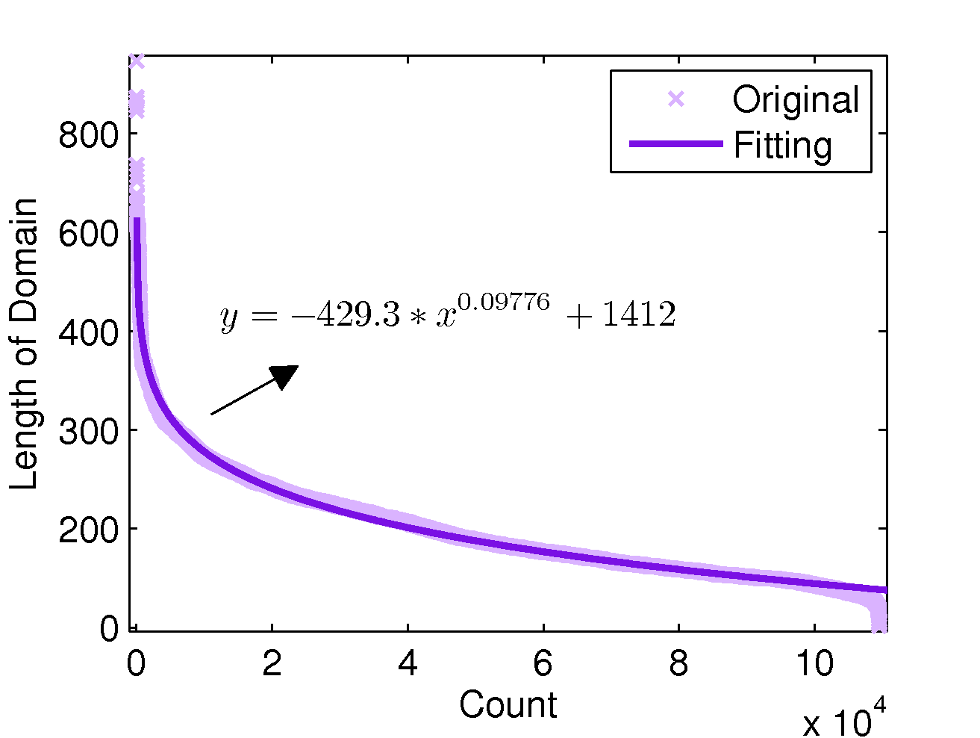

Supplement: Figure S4 — The distribution of domain sequence length. The fitting function is a power law function and the fitting performance is indicated by R-square = 0.9917 and RMSE = 9.93. (TIFF) [file pone.0083788.s004.tiff]

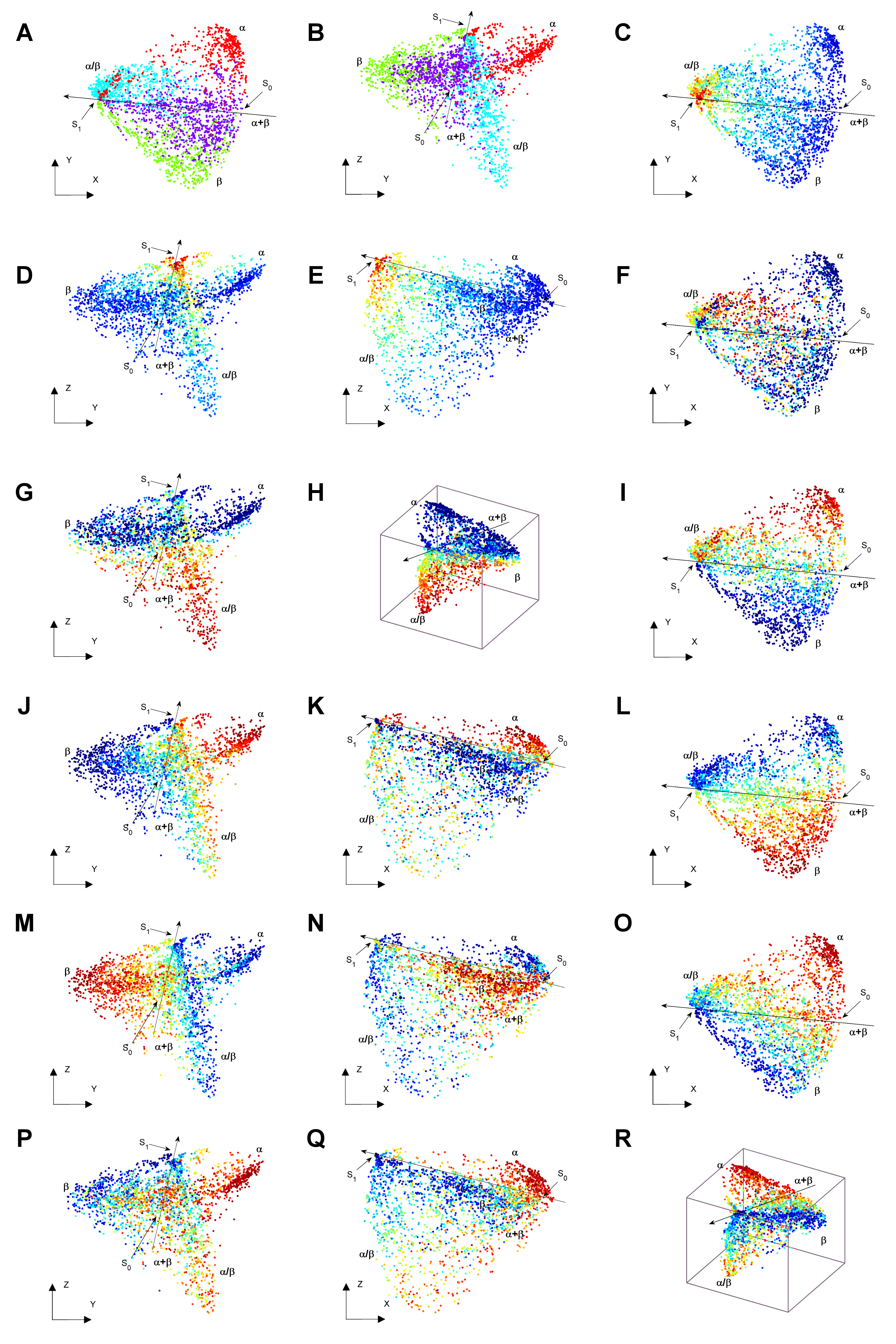

Supplement: Figure S5 — Map of domain structure universe with more perspectives. Class map is shown in X-Y view (A) and Y-Z view(B). Size map is shown in X-Y view(C), Y-Z view(D), and X-Z view(E). Composition map of is shown in X-Y view(F), Y-Z view(G), and a 3-D view(H). Composition map of is shown in X-Y view(I), Y-Z view(J), and X-Z view(K). Composition map of is shown in X-Y view(L), Y-Z view(M), and X-Z view(N). Composition map of is shown in X-Y view(O), Y-Z view(P), X-Z view(Q), and a 3-D view(R). The color schemes are the corresponding ones used in Fig. 4 and described in main text. (TIFF) [file pone.0083788.s005.tiff]

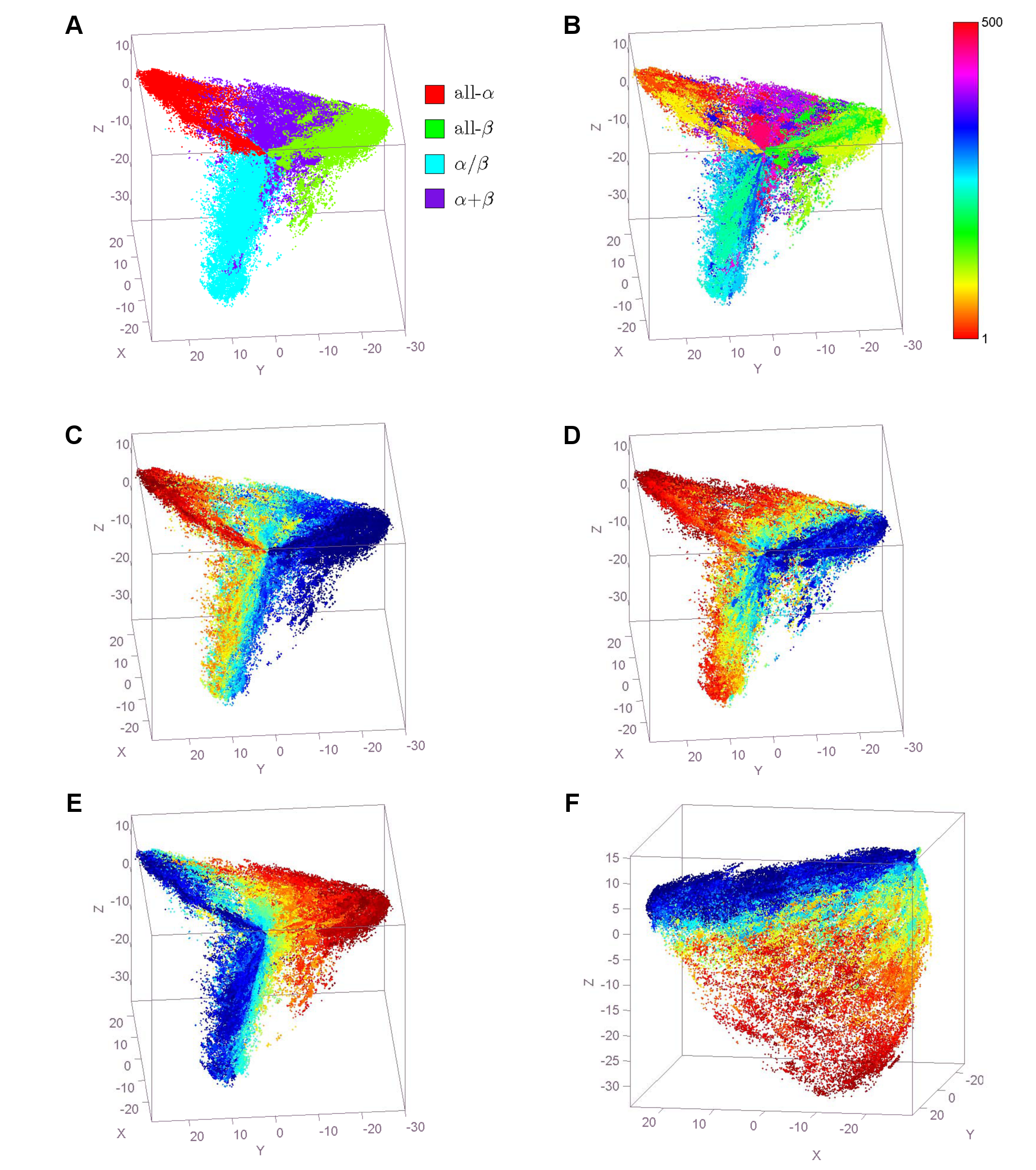

Supplement: Figure S6 — The maps of domain structure universe of SCOP. Totally, 109,533 domains are drawn in maps. (A) In the map of class, red for all-, green for all-, cyan for /,purple for +. (B) Map of 500 Superfamilies. (C)–(F) Map of composition moments: , , and , and the values of composition moments go incrementally from blue to red. (TIFF) [file pone.0083788.s006.tiff]

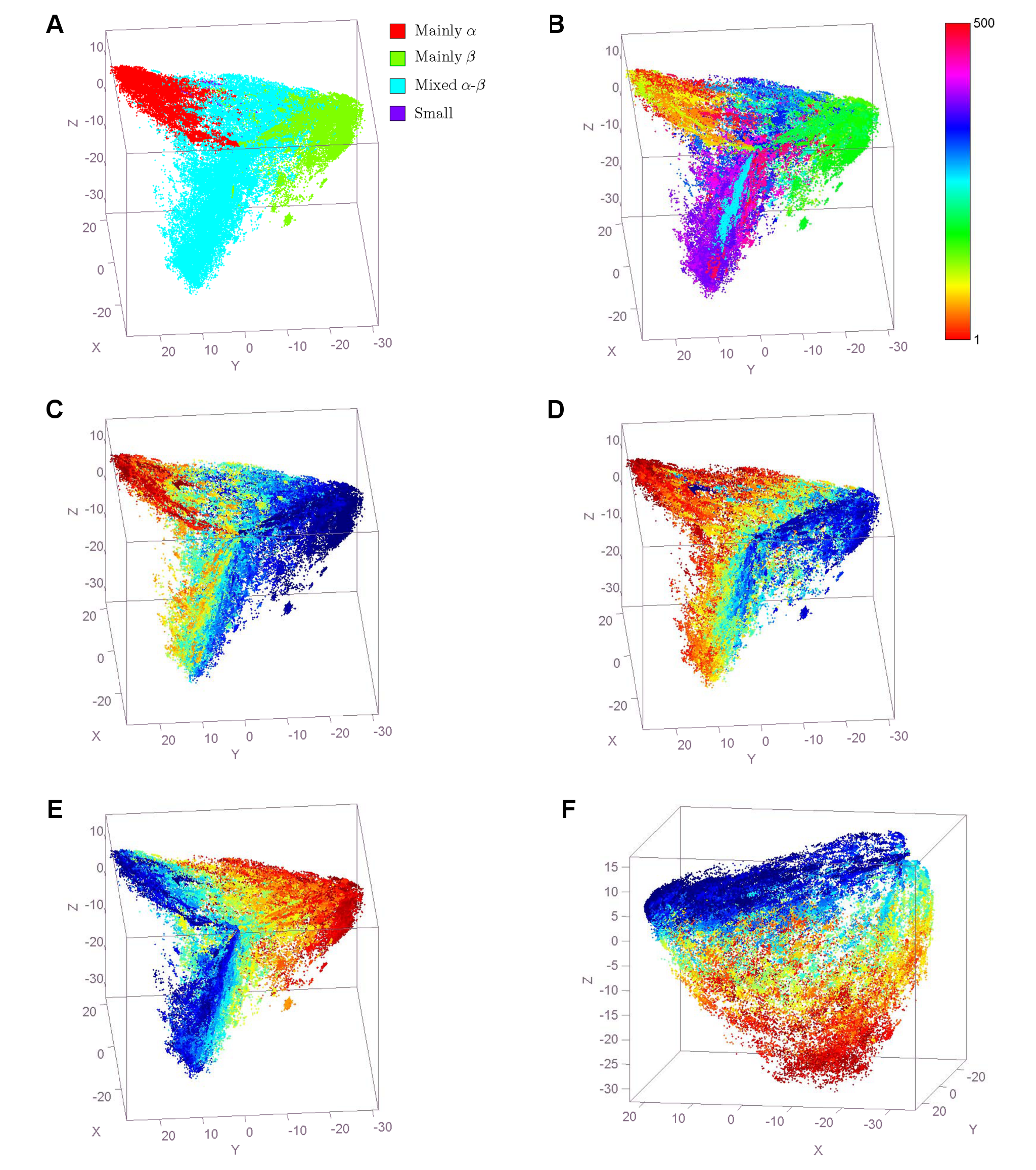

Supplement: Figure S7 — The maps of domain structure universe of CATH. Totally, 98,033 domains are drawn in maps. (A) In the map of class, red for mainly , green for mainly , cyan for mixed -,purple for small protein. (B) Map of 500 Superfamilies. (C)-(F) Map of composition moments: , , and , and the values of composition moments go incrementally from blue to red. (TIFF) [file pone.0083788.s007.tiff]

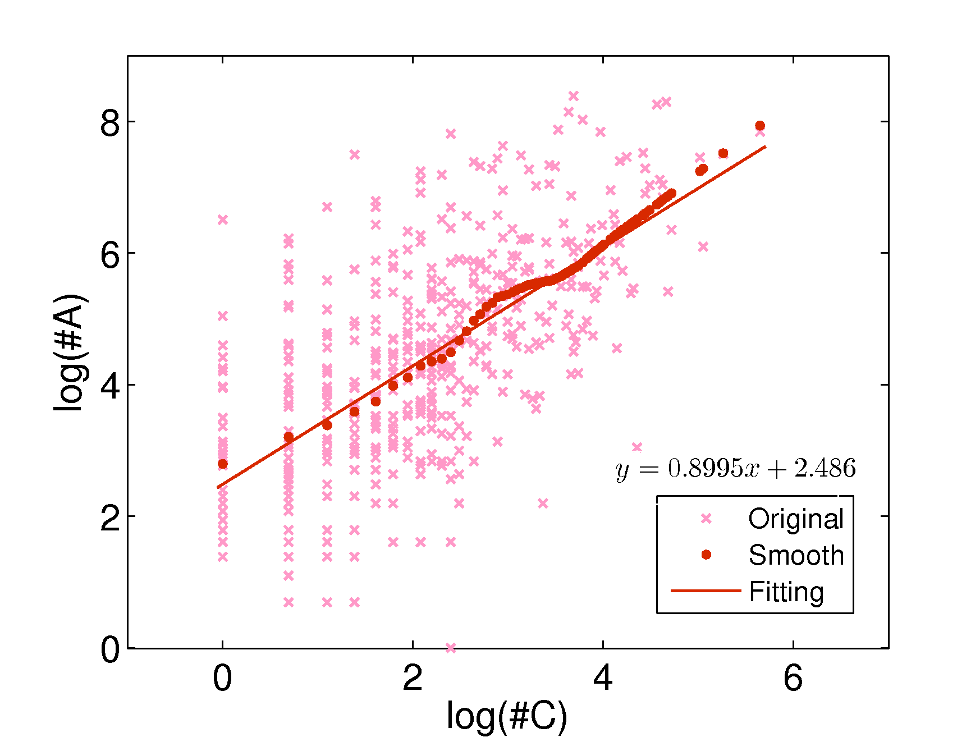

Supplement: Figure S8 — The relationship between the count of clusters (# C ) and the count of unique arrangements (# A ). The logarithmic values of and in selected superfamilies, their smoothed values and the fitting line are drawn together to illustrate the significant linear relationship between and in logarithmic coordinates system. (TIFF) [file pone.0083788.s008.tiff]
