# Supplementary material for: Effective Moment Feature Vectors for Protein Domain Structures
Source: PLoS One. 2013 Dec 31;8(12):e83788. doi: 10.1371/journal.pone.0083788 (PMC3877117; doi:10.1371/journal.pone.0083788)
Supplement: Table S1 — Comparison of classifications on S_H500 with different feature groups. (PDF) [file pone.0083788.s009.pdf]

Tables in SI

Table S1. Comparison of classifications on S_H500 with different feature groups

Feature group*	Dimension	Class (%)	Fold (%)	Superfamily (%)
F0	4	95.2414	78.3505	76.8416
F1	4+9	99.2641	97.7783	97.4929
F2	4+18	99.6293	98.8739	98.6136
F3	4+30	99.7233	99.2170	99.0287
F4	4+45	99.7870	99.3166	99.2058

* F0=$\{m_\alpha, m_{\beta\|}, m_{\beta\perp}, m_{\alpha\sharp}\}$; F1=F0+$\{\lambda_{00}, \lambda_{01}, \lambda_{10}\}_\delta$;
F2=F1+$\{\lambda_{11}, \lambda_{02}, \lambda_{20}\}_\delta$; F3=F2+$\{\lambda_{12}, \lambda_{21}, \lambda_{03}, \lambda_{30}\}_\delta$;
F4=F3+$\{\lambda_{13}, \lambda_{31}, \lambda_{04}, \lambda_{40}, \lambda_{22}\}_\delta$; $\delta = (\beta_\|, \beta_\perp, \alpha_\sharp)$;
$\{\cdot\}_\delta$ means Legendre moments of three binary contact images.
A better result can be expectedly achieved by incorporating higher order moments, however more intensive computation and potential noise will be inevitably integrated.
