# Supplementary material for: Effective Moment Feature Vectors for Protein Domain Structures
Source: PLoS One. 2013 Dec 31;8(12):e83788. doi: 10.1371/journal.pone.0083788 (PMC3877117; doi:10.1371/journal.pone.0083788)
Supplement: Section S1 — Mean shift clustering. (PDF) [file pone.0083788.s010.pdf]

Text in SI

Mean shift clustering

The mean shift algorithm is a non-parametric clustering technique with the advantages of no prior requirement of the number of clusters, no restriction of the shape of the clusters, and easy implementation.

Given a set of data point $\mathbf{X} = \{\mathbf{x}_i\}, i = 1, \ldots, m$ on an n-dimensional Euclidean space $\Re^n$, The so-called "mean shift" is defined as $m(\mathbf{x}) - \mathbf{x}$ at the data point $\mathbf{x} \in \Re^n$, where

$$m(\mathbf{x}) = \frac{\sum_{\mathbf{x}_i \in \mathbf{X}} K_\lambda(\mathbf{x}_i - \mathbf{x})\, w(\mathbf{x_i})\, \mathbf{x_i}}{\sum_{\mathbf{x}_i \in \mathbf{X}} K_\lambda(\mathbf{x}_i - \mathbf{x})\, w(\mathbf{x}_i)} \tag{1}$$

is the sample mean at $\mathbf{x}$, $w(\mathbf{x}_i)$ is the weight of $\mathbf{x}_i$, $K_\lambda(\mathbf{x}_i - \mathbf{x})$ is the kernel function and λ is the radius of region of interest in the given $\mathbf{X}$. The whole procedure of mean shift clustering is described as the following steps:

1. Randomly select a data point $\mathbf{x}^t$ from $\mathbf{X}$ at the beginning time $(t = 1)$,
2. Compute $m(\mathbf{x}^t)$ in the region with the radius of λ at the time t,
3. Translate $\mathbf{x}^t$ to $m(\mathbf{x}^t)$, by $\mathbf{x}^{t+1} = \mathbf{x}^t + m(\mathbf{x}^t)$ at the time $t + 1$,
4. Repeat Step 2 and 3 until $\left\|\mathbf{x}^{t+1} - \mathbf{x}^t\right\| \le \varepsilon$ (The convergence is guaranteed by [35,36]),
5. Set $\mathbf{x}^{t+1} = \mathbf{x}^t + m(\mathbf{x}^t)$ as a cluster centre,
6. Repeat above steps on all data points in $\mathbf{X}$ in turn and assign those data points having same cluster centre with same class labels.

In this paper, in order to speed up the calculation, $w(\mathbf{x}_i)$ is fixed with the value of 1 and the flat kernel with the radius $\lambda = 1$ is used and defined as follows.

$$K_\lambda(\mathbf{x} - \mathbf{x}_i) = \begin{cases} 1 & if\ \|\mathbf{x} - \mathbf{x}_i\| \le \lambda \\ 0 & if\ \|\mathbf{x} - \mathbf{x}_i\| > \lambda \end{cases} \tag{2}$$
